# Understanding the ecosystem of patients with lysosomal storage diseases in Spain: a qualitative research with patients and health care professionals

**DOI:** 10.1186/s13023-021-02168-7

**Published:** 2022-01-14

**Authors:** Juan de Dios García-Díaz, Mónica López-Rodríguez, Montserrat Morales-Conejo, Antoni Riera-Mestre, Minority Diseases Working Group from the Spanish Society of Internal Medicine

**Affiliations:** 1 Clinical Genetics Unit, Department of Internal Medicine, Hospital Universitario Príncipe de Asturias, Alcalá de Henares, Madrid, Spain; 2 Department of Internal Medicine, Hospital Universitario Ramón Y Cajal, Madrid, Spain; 3 Department of Internal Medicine, Hospital Universitario, 12 de Octubre, Madrid, Spain; 4 CSUR de Errores Congénitos del Metabolismo y miembro adscrito colaborador grupo de Enfermedades Mitocondriales Y Neuromusculares, Madrid, Spain; 5 Instituto de Investigación Hospital 12 de Octubre. Centro de Investigación Biomédica en Red de Enfermedades Raras (CIBERER), Unidad 723, Madrid, Spain; 6 Department of Internal Medicine, Hospital Universitari de Bellvitge-IDIBELL, L’Hospitalet de Llobregat, Barcelona, Spain; 7 Faculty of Medicine and Health Sciences, Universitat de Barcelona, Barcelona, Spain

**Keywords:** Rare disease, Lysosomal storage disease, Patient journey, Patient experience, Qualitative research, Quality of life

## Abstract

**Background:**

Lysosomal Storage Diseases (LSDs) are a group of Rare Diseases (RDs) caused by lysosomal enzyme deficiencies. Patients with LSDs suffer from a wide range of symptoms with a strong impact in their daily routines. In this study we aimed to explore the impact of the disease on the lives of patients with four LSDs, as well as how they experience Patient Journey from diagnosis to follow up. Unmet Needs (UNs) perceived by patients and clinicians were assessed to have a better understanding of which initiatives could improve LSDs management and especially those that could result in an improvement of patients’ quality of life.

**Methods:**

Qualitative research was the research methodology selected for the study. It provides plentiful and holistic insights into people’s views and actions. The study was conducted through in-depth face-to-face semi-structured interviews.

**Results:**

In total, 20 patients and 25 Health Care Professionals (HCPs) from different Spanish regions were interviewed. Patients perceived that the highest impact of the LSDs was on their daily routines, specifically on their emotional side, their work/school environment, their family and their social life. Regarding the Patient Journey experience, the worst perceived stage was the pre-diagnosis, where patients only reported negative perceptions, being the delay in diagnosis and misdiagnosis the most commented issues. On the contrary, the follow-up stage was the one with less negative perceptions. Overall, patients and HCPs agreed on the priority UNs, such as accelerating diagnosis, reducing bureaucracy for the treatment access and a more coordinated attention for the patients, not only among different physicians but also with other professionals such as genetic counselors or social workers.

**Conclusions:**

Our data shows that there are still UNs to be addressed from the perspective of patients and HCPs. The main UN is accelerating diagnosis, which could be achieved by medical awareness and education, according to clinicians. A more comprehensive disease management was another main point to be worked on to improve LSD-patient experience and quality of life.

**Supplementary Information:**

The online version contains supplementary material available at 10.1186/s13023-021-02168-7.

## Background

Rare diseases (RDs) are defined in the European Union (EU) as the ones that affect no more than 1 in 2000 people. This suggests an estimate of up to 36 million people affected in the EU given the RDs identified to date [1]. Lysosomal Storage Diseases (LSDs) are a group of RDs that share a deficiency in a lysosomal enzyme which leads to the storage of the defective-enzyme substrate. Depending on the specific enzyme affected, different molecules will be accumulated. In all cases, the storage triggers lysosomal and cell disfunction per se, as well as the activation of signalling pathways with added deleterious long-term effects, such as inflammatory pathways [2, 3]. LSDs are inherited and debilitating metabolic disorders, with a wide range of multiorganic clinical signs and symptoms that progress at variable rates [4].

Pinpointing the molecular defect underlying in a RD is not always easy. LSDs were among the first RDs in which the cause of the disease could be linked to an alteration in an enzyme [4, 5]. A group of misfunctioning enzymes, all confined inside the lysosome, were giving raise to LSDs. The classification of LSDs has been based on the nature of the accumulated molecule due to the missing or reduced enzymatic activity. For example, glycogen is stored in Pompe Disease (PD), mucopolysaccharides in mucopolysaccharidosis (MPS) while glycosphingolipids of different types are accumulated in Gaucher Disease (GD) and Fabry Disease (FD) [4, 5]. Depending on the degree of enzymatic activity reduction, the number and kind of mutation/s, among other modifying factors yet to be identified, the symptoms may arise in the paediatric-age or later in life, with variable organ affection and severity. The early identification of the pathological pathways involved, prompted a much deeper scientific knowledge of these four LSDs in comparison with other RDs. Yet, the experience with people with LSDs is scarce in the published literature [6].

Qualitative research explores complex phenomena encountered by clinicians, health care providers, policy makers and consumers in health care. Interviews are widely used as a data collection tool in qualitative research [7]. They are typically used as a research strategy to gather information about participant’s experiences, views and beliefs concerning a specific research question or phenomenon of interest. The semi-structured interviews offer a more flexible approach for the interview process. While they may use an interview schedule for determined topics, they allow for unanticipated responses and issues to emerge using open-ended questioning [8].

Therefore, qualitative research based in semi-structured interviews was the research vehicle selected for our project. Thus, we aimed to analyse the experience of patients with PD, MPSI, FD and GD during all their journey, from pre-diagnosis to diagnosis, follow-up, control of the disease and quality of life, and healthcare specialists’ perceptions.

## Methods

### Study design

To set up this study, a group of internal medicine clinicians with broad experience in LSDs, organized into a Scientific Committee to decide the guidelines of the research and what qualitative research method was best to address the goals of the study. As such, the committee set the project guidelines, discussed the characteristics of patients and physicians needed to have a representative sample, and the specific elements of the Patient Journey to be studied more in depth.

A Medical Agency (MA), Anima Strategic Consulting, with expertise in the matter, was also chosen. After several meetings around needs and goals, the study design, protocols, and materials (interview guides, card sorting, etc.) were set by the Scientific Committee and the MA.

**Fig. 1:**
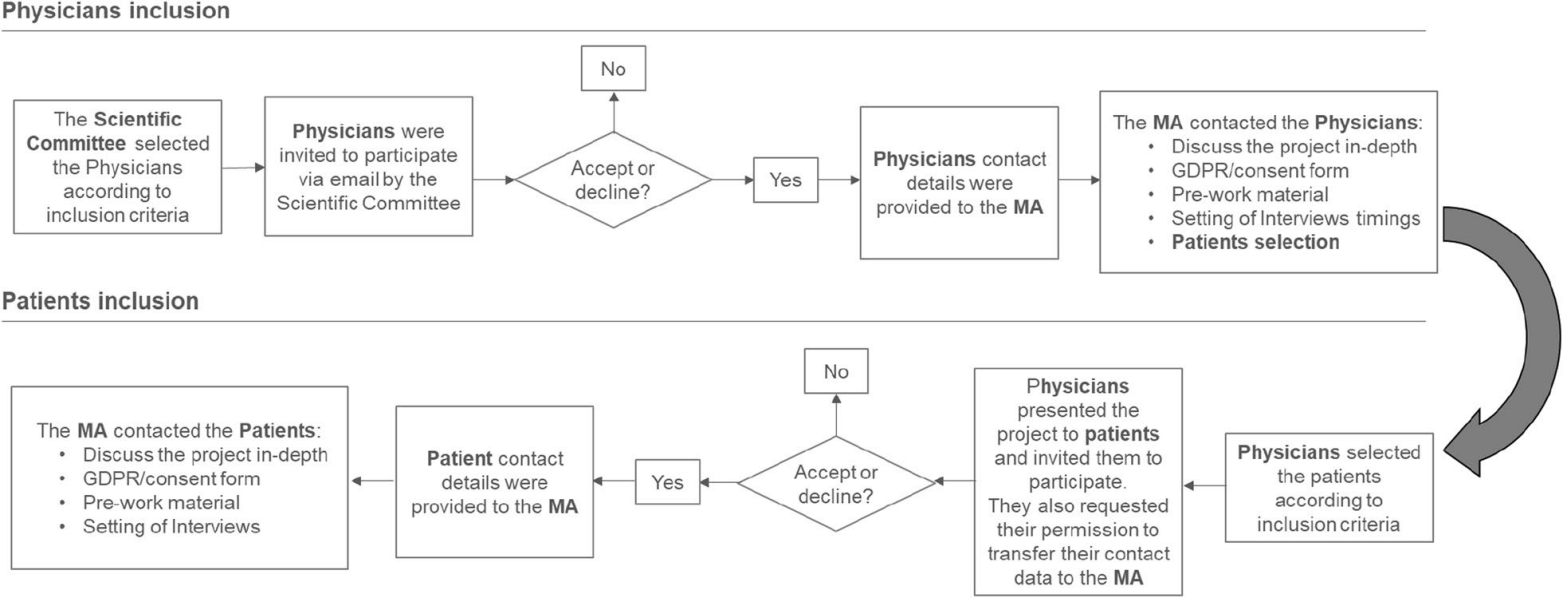
Diagram depicting the selection process of clinicians and patients. MA: Medical agency

The Scientific Committee selected and invited to participate a group of clinicians based on the criteria described below. These clinicians were responsible for the selection of patients, based on the criteria also described below. As the objective of this qualitative study was to explore the impact of the disease on the lives of individuals with LSDs all along the Patient Journey, as well as to assess the UNs of patients and clinicians throughout the care process, in-depth interviews with both patients and HCPs were performed (Fig. 1).

### Sampling of clinicians and patients

To recruit clinicians and patients, a non-probabilistic purposive sampling with pre-established inclusion criteria was used. To have different backgrounds and circumstances, the sample of patients had to include patients with and without specific treatment, from different geographical Spanish regions and from rural and urban areas. Five patients diagnosed with each one of the mentioned LSDs (FD, PD, GD and MPS I) were selected.

In the case of the selected clinicians, they all had to have at least 5 years of professional experience as specialists and at least one year of LSDs-specific experience and be familiarized with all the stages of the Patient Journey. To include a variety of views, the sample had to include diversity on hospital size, HCPs from RDs-reference and non-reference centres as well as geographical dispersion across Spain.

### Research instrument

The qualitative research was carried out via semi-structured interviews from September 2018 to December 2018. Prior to the interview, pre-work assignment with questions regarding the most relevant aspects of the interview was provided to the interviewees to make them reflect on relevant aspects of the interview. Pre-work was a reflexive exercise, the individuals with LSDs and HCPs wrote their reflexions, and this material was analysed in detail during the interview. The questions addressed in the pre-work assignment are detailed in Additional file 1: Table S1.

- In the case of patients, the reflection was oriented towards their experience with the disease, from the moment they started experiencing symptoms to follow-up, including the moment they were diagnosed and began treatment. Perceived UNs were also commented.
- In the case of physicians, the reflection was oriented towards their priorities in managing people with lysosomal diseases, as well as the characteristics and needs of their patients.

The in-depth interviews carried out were based on two semi-structured scripts -one for patients and one for HCPs-, with an approximate duration of 90 min. Semi-structured interviews allow the generation of rich, in-depth data, while using an interview guide to direct the content [10].

All the interviews were carried out face-to-face. The location of the interview was chosen by the interviewees (i.e., in a cafeteria, at patient's home, at hospital), which allowed to create a better environment for them. In the case of the clinicians, all the interviews took place in their offices. The two interviews with underaged patients in MPSI were conducted in the presence of their parents.

The scripts were organized in five different sections, four of them common for patients and HCPs and one specific section for each. Diagnosis, treatment, follow-up, and ways of improving quality of life were the common sections, while professional profile was included only for HCPs and impact of the LSD on daily routines only for patients. A summary of the interview guides are detailed in Additional file 2: Appendix A and Additional file 3: Appendix B.

Interviews were complemented with reflective/projective techniques to better capture insights. A Card Sorting technique was used as a complement. HCPs and patients were given a series of labelled cards, were asked to organise them into groups, and to sort them by the degree of importance. The number of cards determined the minimum and maximum prioritization score. The cards used in the card sorting exercise are detailed in Additional file 1: Tables S2 and S3.

The Consolidated Criteria for Reporting Qualitative research was used to ensure that our study meets the criteria for qualitative research [7].

### Data collection and analysis

The interpretative description was the methodology guiding this study. Interpretative description does not require using a theoretical framework per se, but rather is a pragmatic methodology focused on generating findings that are useful for clinical practice [9].

All semi-structured interviews were recorded and transcribed verbatim. The data was analysed using a constant comparative and iterative approach, reading, and comparing transcribed texts to determine if they reflected the same concept or theme. Transcriptions were analysed by three independent researchers by the MA. The researchers inductively and independently built categories according to the study aim. The three researchers read all the transcripts and hand-coded the data. These codes were discussed, revised, and compared collectively to refine the coding structure by adding codes as new insights were gained. These new themes were verified during subsequent interviews, asking more aspects in some questions introduced by the interviewer. This process helped to improve the rigor and validity of the analysis.

The final coding was discussed with an additional researcher from the MA until reaching an agreement during a meeting that followed, to further discuss the main findings of the research and to reach a consensus in case of discrepancies.

To classify the priorities using the card sorting technique (i.e., high–medium–low impact, high–medium–low importance), the analysis was performed by computing the average of each individual outcome. With all the obtained information, the Scientific Committee and the MA worked through the results to perform a deeper analysis.

## Results

### Clinicians and patients characteristics

Overall, 20 patients and 25 HCPs scattered all around Spain were interviewed. The characteristics of the participants are described in Table 1. Although the focus of this work was the patient´s experience, more HCPs than patients were included to facilitate the inclusion of patients according to the inclusion criteria.

**Table 1:** Number and characteristics of patients and clinicians

	LSD	FD	GD	PD	MPSI
Patients	Number of patients	5	5	5	5
	Sex	Male (4)	Male (3)	Male (2)	Male (1)
		Female (1)	Female (2)	Female (3)	Female (4)
	Age				
	Average years (SD)	45.0 (4.0)	6.8 (17.9)	0.0 (16.5)	29.0 (20.4)
	Median [Min–Max]	46 [40–50]	65 [26–70]	51 [30–68]	21 [9–58]
	Diagnosis time from the first visit				
	Average years (SD)	8.9 (9.8)	4.8 (7.1)	17.6 (12.4)	12.1 (16.0)
	Median [Min–Max]	5.0 [0.2–20]	2.0 [0–15]	19.5 [1.5–30]	7.1 [0–34]
	Specific Treatment	Yes (5)	Yes (4)	Yes (4)	Yes (3)
			No (1)	No (1)	No (2)
Clinicians	Number of Physicians	9	6	5	5
	Medical Area- Speciality	Internal Medicine (3) Nephrology (3) Cardiology (3)	Internal Medicine (4) Haematology (2)	Internal Medicine (2) Neurology (3)	Internal Medicine (3) Paediatrics neurology (2)
	Years of experience in LSDs				
	Average (SD)	12.2 (6.5)	14.2 (8.7)	7.4 (3.9)	15.8 (7.0)
	Median [Min–Max]	11 [4–25]	15 [5–25]	8 [1–11]	16 [8 – 25]

FD: Fabry disease; GD: Gaucher disease; PD: Pompe disease; MPSI: mucopolysaccharidosis type 1

Medical specialties were in accordance with the signs and symptoms of the given LSD. In the case of GD, all patients were affected with Type 1, in PD all patients were late-onset phenotype, and in MPSI, all patients had the mildest type: the Scheie Syndrome. Sixteen (80%) out of 20 patients were receiving treatment at the time of the study.

### Impact of the disease on the habits and routines of patients

We analysed the different perception of patients on the impact of the LSD on their daily routines. Patients would range the impact with labeled cards. A brief description of signs and symptoms, together with the course of each LSD is included in Table 2 to facilitate the reader´s understanding of the clinical course of these pathologies. Patients with these four LSD have very different clinical symptomatology. Moreover, there is also a high variability within a given LSD. In general, patients end up with physical activity restrictions due to the disease.

**Table 2:** Description of the signs and symptoms, and the course of included LSD

LSD	Signs and symptoms	Course of the disease
FD	Main symptoms at onset: fatigue, pain, fever crisis, digestive discomfort, heat stroke, pain in the extremities, foam in the urine, etc Early presence of symptoms in paediatric age	Shortened life expectancy and a significant loss of quality of life FD progression in target organs such as heart or kidneys, such as kidney failure, cardiomyopathy, and cerebrovascular ischemic events (stroke) with irreversible consequences for the patient
GD	The most common sign is a swollen spleen, which can be followed by bone disease and brain involvement and other problems such as tiredness, bleeding, bruising, and lung disease Pain due to bone or neuronal involvement is the most common reason for consultation Thrombocytopenia and anemia	Degenerative disease that can progress to increased visceromegaly (hepatomegaly and splenomegaly), recurrent bone pain, and lung involvement
PD	Symptoms little visible at onset with low impact on quality of life initially The first symptoms are: muscle weakness and difficulty walking, breathing problems and infections of the respiratory system and failure to gain weight and growth at the expected rate. HyperCKemias	Muscle weakness (which can make it very difficult to walk), and breathing problems, that will finally lead to the use of walking aids and mechanical ventilation
MPSI	In the first years of life, patients present visible musculoskeletal alterations of varying degree, as well as corneal opacity	Very disabling degenerative disease that affects children in its most serious forms High impact on quality of life due to physical limitations and cardiovascular and respiratory complications

These four LSD have a wide range of clinical presentation and different phenotypes exists

FD: Fabry disease; GD: Gaucher disease; PD: Pompe disease; MPSI: mucopolysaccharidosis type 1

The greatest impact patients mentioned were related to their “emotional side”, their “work/school environment”, their “family”, and their “social life”. The results of this investigation are described in Table 3.

**Table 3:** Impact on habits and routines of patients and examples of comments about the impact of the disease

High impact	**Emotional**	When they tell you that you are sick, your environment is very understanding about your illness and you feel protected, but over time they no longer try to understand you, they just feel sorry…and that is emotionally very hard. [FD]
	Feeling helpless	
	Difficult childhood	
	Concern with contradictory information (on-line, RSS)	
	**Work/school**	I had to quit my job because I got really tired and could not keep up. It was hard for me to hit the pedals of the sewing machine. [PD]
	Inability to carry out some work/school related activities	
	Inability to get a steady job	
	Inflexibility to adapt to work/school schedules	
	Ignorance/Difficulty to get the disability retirement or social benefits	
	**Family**	I was so worried about my son. When they did the tests and they told me that he was also ill, I thought that he would not forgive me. [FD]
	Feeling of guilt owed to the possibility of genetic transmission	
	Deciding to have children or not can cause problems in the couple. Limitations to carry out daily activities with their partner and children	
	Impossibility of traveling or spending a period of time (i.e. vacations) away from the hospital	
	**Social**	When I was a teenager, I do remember that I had such a big belly that it attracted attention and people would stare at me, thinking «look at this girl so young and already pregnant». It was hard at the time and I even stopped going to the pool. [GD]
	Lack of understanding on the LSD-burden when there is absence of a differentiating external phenotype	
	Yet, being the target of jokes or contempt by others in patients with an obvious different phenotype	
Medium	**Stop doing things**	I wish I could open a bottle or not being so dependent on my mother constantly. I have always had my hair short, so she does not have to comb my hair. [MPSI]
	Stop doing daily activities such as doing sports, walking long distances, carrying shopping bags, etc	
	**Exercise/physical therapy**	I should do more, but the truth is that walking to go to work or to see my brother involves physical exercise and you cannot force the machine that much. [MPSI]
	Restricted physical activity	
	Having to perform less aggressive activities (i.e., walks, swimming, dancing)	

FD: Fabry disease; GD: Gaucher disease; PD: Pompe disease; MPSI: mucopolysaccharidosis type 1

Their disease manifested in their day to day to different extents depending on the onset or severity of symptoms and not always on organic damage. Patients with FD or MPSI saw their quality of life more limited. On one hand, Fabry patients had suffered pain crisis, GI-symptoms and fatigue during their childhood that had very frequently conditioned their adult behaviours with insecurities at work or other social environments. On the other hand, patients with MPSI, with visible physical symptoms -mainly skeletal deformities-saw their quality of life limited, mainly due to mobility difficulties as the disease progressed. For most individuals with LSDs, the inability to carry out some activities at work or at school, sometimes limited the education and career opportunities. However, less limited patients could perform physical activities, sometimes following their doctors’ recommendations.

Regarding the family impact, in some cases, they felt incomprehension from their close environment concerning some symptoms such as fatigue, which on many occasions changed to acceptance after diagnosis. Nevertheless, patients generally felt understood and supported by their closer family.

### Patient journey

The Patient Journey was elaborated based in the perceptions patients had. Individual perceptions are collected in Table 4.

**Table 4:** Patient journey positive and negative aspects from the patient perspective

Pre-diagnosis	Diagnosis	Treatment	Follow-up
♦ Annoying symptoms, but difficult to associate with LSD, especially when there is no organ involvement (i.e. fatigue, fever, respiratory difficulties, etc.) ♦ Continuous referrals to different HCPs until reaching the expert who would suspect an LSD ♦ Misdiagnosis ♦ Years pass by before reaching a diagnosis	♣ Relief of having a diagnosis that explains the symptoms ♣ Once there is suspicion of the LSD, appropriate access to testing ♣ Information transferred by the doctor gradually is perceived favourably ♣ Slow but relieving assimilation process: there is no cure but there is a treatment to attenuate the development of the disease ♦ Patient overwhelmed due to the high number of tests to be performed in occasions to reach a final diagnosis ♦ First time ever for patients to hear about their disease ♦ Difficult to learn of the progressive and limiting symptomatology ♦ Uncertainty about further details related to the disease ♦ Information overload on the Internet about the disease ♦ Emotionally affected when learning about the possibility of genetic transmission of the disease	♣ Reliance on the specific specialist ♣ Information about treatments delivered on the right timing ♣ Great expectations in treatment efficacy ♣ Huge emotional support provided by the nursing care service ♣ Great content with home therapy ♦ Red tape for treatment approval initiation ♦ Prolonged switch of treatment procedures ♦ Long waiting time during treatment administration ♦ High hospital dependence ♦ Uncertainty about treatment response of the patient	♣ Appropriate and satisfactory follow-up in terms of doubts resolution, carried out by the physician of reference ♣ Frequency of control and follow-up visits perceived as appropriate ♣ Excellent coordination among the healthcare specialists to carry out complementary testing ♣ Appropriate access to complementary testing ♦ Lack of knowledge of the disease by other physicians, especially GPs ♦ Difficulties arising from holidays organization or requesting recurring sick leaves

♦: Negative aspects; ♣: positive aspects

The only stage in which patients only referred negative aspects was in the pre-diagnosis. The delay in diagnosis and misdiagnosis were the most mentioned issues. In MPSI patients, with a marked external phenotype since childhood, reaching diagnosis generally took only a few months in contrast to much longer diagnosis times in the other LSDs. Yet, a MPSI patient is the one with a greater diagnosis delay in our sample, although the reason for this delay was not identified.

In general, once the diagnosis was reached, patients felt comfort in finally naming their condition and relief to know there was a treatment that attenuated the progression. Nevertheless, they also felt disappointment when they learnt their disease was progressive and incurable. The patients’ experiences differed depending on whether the diagnosis was reached as a child or as an adult. Those patients diagnosed as adults reported more negative aspects regarding pre-diagnosis and diagnosis stages, due to the diagnostic delay. One of the main concerns referred by patients was the possibility of transmitting the disease to their offspring, to the point that even guilt was present in parents of affected children.

Regarding specific treatment and follow-up, both paths were parallel in patients who started with the first one. Some LSDs have oral or intravenous treatments with different degree of published evidence, while others have only an intravenous treatment. On addition, patients had to attend a close and multidisciplinary follow-up. Therefore, hospital dependency was elevated, and sometimes hospital visits interfered with personal life.

Regarding treatment, most patients showed confidence in their specialists. Once the decision of starting treatment was made by the patient and the specialist, a long bureaucratic process started before approval, being the final decision taken by a committee. Patients expressed surprise, indignation, annoyance or understanding for not being examined directly by these committees.

All patients felt empowered and satisfied when the treatment started, hoping their limitations would be reduced, but also faced uncertainty regarding their treatment response. The latter was more marked for many PD and MPSI patients since there is only one therapeutic option.

The follow-up stage was the one in which there was fewer negative perceptions referrals. All patients had a close relationship with their LSD-specialist physician. A special mention needs to be made about the feelings of parents of LSD-patients and of those patients diagnosed at childhood. The moment of transition from the care of the Paediatrician to the follow-up by Internal Medicine specialists was anticipated by parents and experienced by patients with uncertainty and anguish. This was due to the fear of losing the bond with the Paediatricians after many years of building a strong relation with this specialist.

### Clinicians’ priorities on lysosomal diseases

Regardless of their specialty, background, and specific LSD with which they were involved, the first priority for physicians was early diagnosis of the LSD, as this was a *sine qua non* condition for early management of patients. For over 40% of clinicians, alleviating and controlling symptoms to delay organic damage was another of their priorities. Approximately around 40% also mentioned “maintaining the patient’s quality of life” as another of their priorities, “avoiding any type of irreversible complication” and “identifying other possible comorbidities”.

Although main medical priorities were shared by HCPs independently of the specific LSD, there were priorities adapted to the course of the LSD. For example, in PD an early treatment allows preserving the motor and respiratory functionality, thus postponing or avoiding wheelchairs and mechanical ventilation. For these patients, that lacked evident symptoms initially, early treatment was precisely the main medical priority for HCPs. Table 5 gathers HCPs´ priorities within an LSD.

**Table 5:** HCPs priorities by LSD

LSD	Priority #1	Priority #2	Priority #3	Priority #4
FD	Alleviating and controlling symptoms	Early diagnosis to relatives	Strict follow-up of patient symptoms	Emotional care of the patient
GD	Avoid irreversible complications	Detect neurological deficits	Strict follow-up of patient symptoms	Keep the patient informed
PD	Early start of treatment	Preserve patient functionality	Maintain the patient’s quality of life	–
MPSI	Maintain the patient’s quality of life	Optimise visits to the hospital	Give a real vision of disease evolution to the patient	Performing bone marrow transplant to paediatric patient

FD: Fabry disease; GD: Gaucher disease; PD: Pompe disease; MPSI: Mucopolysaccharidosis type 1

### Unmet needs

We analysed what patients and HCPs perceived as UNs. The UNs perceived by patients and HCPs were categorized in three groups: “Medical Attention” (diagnosis, HCP expertise and coordination between different HCPs), “Treatment” (access, ease of administration, scientific research) and “Disease Management” (information and awareness, patient’s comprehensive approach, and specific solutions for the improvement of quality of life). Some individual perceptions are collected in Table 6.

**Table 6:** Main needs of specialist and patients around lysosomal diseases

Scope	Main need	Importance	
		HCP	Patient
Medical attention	Improving the diagnosis	10	9
	Awareness among specialists is very important to speed up the diagnostic processes and to treat as soon as possible. This dissemination should start in the RD specialty centers/Units [HCP]		
	HCP medical awareness and education	10	6
	Sometimes the practitioner that meets these patients does not have enough experience or assertiveness. When physicians [with no LSD-expertise] have one of these patients, they don´t know, who to turn to or what to do. [HCP]		
	Coordination between HCP	8	5
	The case manager oversees all the process ensuring that all steps are done correctly, that patients don’t have duplicate and unnecessary visits. A case manager is obsessed with simplifying all channels and patient routes. And we do not have this figure at the moment… [HCP]		
Treatment	Access to treatments	9	9
	We tried very hard to get the medication approved for him [our son]. And not only us, also his doctor. I wondered «Will it be necessary for him to have a damaged organ so that they finally decide to approve his treatment?» [Patient]		
	Ease of administration	9	9
	We are in love with Home Therapy. It is overwhelming, patients are very happy that they do not have to travel, and it represents a saving of around €60,000 per year. And despite that, it is delayed by the Administration. [HCP]		
	Scientific research	9	5
	In 3–5 years, we have seen more progress than in the last 20. But the future of these pathologies lies in gene therapy, there is still a long way to go. [HCP]		
Disease management	Information and dissemination about de disease	8	9
	It would be necessary to have a communication channel with the doctor, by whatsapp, or an application that if something happens to you, you can consult them, especially if what happens to you is something important. [Patient]		
	Comprehensive approach of patients	10	9
	When there is swelling of the legs due to a malfunction of the lymphatics, a specific lymphatic physio is very difficult to access. They are overwhelmed with work and highly sought after. [HCP]		
	Specific solutions to improve patient’s quality of life	8	10
	It is a limitation. For example, picking things up off the ground, it seems silly, but it involves an effort that is often complicated for me. I have to keep the thongs in sight because sometimes it helps. [Patient]		

Level of importance and examples of comments about these needs. Importance: 1 low–10 high

#### Medical attention

Patients and specialists perceived early diagnosis as a very important UN. For HCPs, defining and standardising diagnosis criteria within clinical guidelines would be critical to ensure early diagnosis. Nevertheless, HCPs agreed on the fact that family testing, screening programmes as well and the development of Dried Blood Spot tests, had significantly improved early diagnosis. On the other hand, specialists emphasized on the need of patient-centricity, which implied coordination between the medical multidisciplinary team and social care experts.

#### Treatment

HCPs considered important to homogenize treatment-access-criteria among regions as well as reducing administrative procedures, which was also a strong UN for patients. On the other hand, for HCPs was very important promoting scientific research focused on expanding therapeutic options, especially in the diseases with a single treatment option (PD and MPSI). Clinicians had great expectations in gene therapy as a way of reaching a final cure. Additionally, the existence of industry-independent registries was perceived as a means of improving knowledge of long-term outcomes. For patients and physicians improving patient experience during infusions was also important, for HCPs this meant separating LSD-patients from other seriously ill patients, reducing waiting times, and journeys to the hospital by promoting home therapy.

#### Disease management

HCPs demanded a better promotion at national level of the existence of RDs-speciality centres/Units, as well as more resources and clearer boundaries at the political level of the capabilities and responsibilities of such structures. For both HCPs and patients, an UN was an easier access to genetic counselling and psychologists. Many patients also mentioned the need of social workers, as they often lacked information about the governmental benefits they were entitled to. Patients and HCPs also missed having reliable and verified sources of information adapted for patients. On the other hand, clinicians demanded forums where to debate on clinical cases with RD-specialists. The transition of paediatric patients from paediatricians to adult RDs-specialized HCPs was also an important UN for patients and parents. A better understanding by Society was also an important UN.

## Discussion

To the best of our knowledge this is the first qualitative study to be published in a peer-review journal with LSD-patients in Spain. We decided to include a wide range of divergent characteristics of patients within the four LSDs to have different views and perspectives. There was a mixture of patient profiles which included phenotypical variation and geographical dispersion.

This study has deepened in the way individuals with four LSDs experience their journey with the disease. Using qualitative research as the means to evaluate the relation these patients have within their “ecosystem” of self, family, medical attention and Society. These four LSDs share a common scientific background and the availability of a specific treatment for almost 20 years.

### Impact and pre-diagnosis

The impact the LSD had on the lives of the patients in our study are common to the ones described in many other qualitative RD publications. Physical impairment, pain, fatigue, and psychological burden have a toll on day-to-day activities, on career opportunities and on family activities in RD patients [10–12]. Concerns around family planning and feelings of guilt due to the possibility of transmitting the disease are also a common trait in RD qualitative studies [12, 13]. Lack of understanding by society, was felt by the patients in this study to different degrees and at different time-points in the evolution of their disease, which is also frequently mentioned in the literature in relation to RDs in general [12–14].

### Diagnosis

Misdiagnosis was frequently mentioned in our study which is also described in the literature. As an example, different publications have explored wrong diagnosis given to Fabry in paediatric [15] and adult patients [16], and are a good example of the challenge that supposes the diagnosis of such infrequent diseases with an ample clinical spectrum. Regarding the time from the onset of symptoms to diagnosis, our study shows an average of five years delay, with one patient needing 34 years for a final diagnosis.

One of the main issues and of the main priorities referred by all physicians and patients in our research was the importance of early diagnosis. In the literature, delays of up to five years in MPSI Scheie, ten in GD Type 1 and 16 for PD have been reported [17–19]. A European study assessed the delay between symptoms-onset and diagnosis in FD in two consecutive periods of five years, from 2001 to 2013. The average diagnosis delay decreased from 18 to 13.3 years [20]. Indeed, other qualitative studies on RDs have explored diagnosis delays. A Spanish qualitative study on RD [21], concluded that 21% of patients had had diagnosis delays of ten years or more, although their data also reflected shortening of waiting times in recent years. So, although improvements are being made in early diagnosis, there is still much to improve in the view of patients and of physicians, as they both ranked diagnosis in the top-three UNs.

Medical suspicion and family testing are the most effective ways of early diagnosis, followed by screening programmes. Congruently, clinicians specifically prioritized medical education to improve early diagnosis, being primary care education especially important in their opinion. Medical unawareness has also been cited by patients and physicians as one of the causes leading to diagnosis delays and mistreatment in other qualitative RD studies [11, 13, 21]. In Spain, a study published in 2020, evaluated by surveys the education received by specialists and primary care physicians on RDs. 27% of all the surveyed HCPs recalled having had specific education on RDs on their medical degree and around 40–45% as continuous-medical-education in the last five years [22].

For the patients in this study, the diagnosis moment was a contradictory moment because of the sense of relief for finally naming their medical condition, yet it mixed with uncertainty feelings because of what could await ahead and because of the lack of enough reliable information. In the work by Esquivel-Sada [13], 2018, diagnosis is a moment of empowerment for RD patients, yet uncertainty regarding the course of the disease and not sufficient reliable information on the pathology are also described in the literature [11, 12, 21]. Indeed, quality information for patients was classified as a main UN both by patients and HCPs in our study.

### Treatment and follow-up

All patients in our study felt relief when they learnt there was a specific treatment for their condition, aware that this is not the case for most RDs. As reviewed by Lippe et al. [6], several publications describe how some patients feel fortunate for living in a geographical area with available treatment options. As published by Kesselheim et al. [23], some patients and caregivers with RDs and no available treatment expressed they would take any “medication” option, even alternative therapies. Again, for many patients in our group, expectation together with fear and uncertainty mixed regarding response to the available treatment.

In our research, all patients had a very good relationship with their specialist which is also common in many RD studies [11, 13] but not in all [21, 23]. Nevertheless, the positive relation with the specialist in our study might be biased by the used methodology of patient selection.

Follow-up was burdensome for most of our patients, due to the multiorganic features of the selected LSDs. Multi-organic affection is shared by many RDs, and many patients complaint on the many different tests they must take periodically, and how coordination among HCPs is many times improvable [12, 13, 21, 23].

### Study strengths and limitations

This study has some limitations and strengths that must be mentioned. A bias inherent to surveys and qualitative studies is the interviewee or acquiescence bias, in which respondents tend to select a positive response/connotation disproportionately more frequently [24]. Nevertheless, qualitative methodology is a method for gaining deeper insights into UNs and it is ideally suited for investigating the key psychological, emotional, and social specific aspects of living with a rare disorder (attitudes, quality of life impact and motivations). Carrying out the research in their own environment, observing them and accompanying them in their routines also helped identify non-explicit aspects that also affected their habits and attitudes, and it allowed for a better understanding as to how they lived with their disease and how they interacted with the different agents influencing their care and treatment. Many common wordings were present in our study and in the comprehensive review by Lippe et al. [6], on qualitative research in RD. So, the patients experience seems not to be local, at least in Europe and North America, therefore, although one of the limitations of our study is the small size of the sample, our results are consistent with published data on qualitative studies involving RDs [6, 10–14, 21, 22].

## Conclusion

In conclusion, our data reflect that there are still UNs to be addressed both in the view of people with LSDs and HCPs. For both patients and clinicians, accelerating diagnosis is crucial, being medical awareness and education the main paths to its achievement for clinicians. A comprehensive disease management was another main point to address patient’s quality of life that could improve LSDs-patients’ experience. Knowing first-hand the UNs of both patients and HCPs is essential to define new forms and initiatives that would help to respond to these needs and improve the satisfaction of all those in the ecosystem of the LSDs, especially the people with LSDs.

## Supplementary Information

**Additional file 1.** **Table S1**. Pre-Work assignment sent to patients and clinicians prior to the in-depth interview.** Table S2**. Card Sorting Technique used during the interview with patients.** Table S3**. Card Sorting Technique used during the interview with clinicians.

**Additional file 2. Appendix A**. Patients’ Script Summary of patient’s in-depth interview: a full description of the questions usedfor the patient’s in-depth interview.

**Additional file 3. Appendix B**. Physicians’ Script: Summary of clinician’s in-depth interview: a full description of the questionsused for the physicians’ in-depth interview.

## Data Availability

The datasets used and/or analyzed during the current study are available from the corresponding author on reasonable request.

## Abbreviations

EU: European Union
FD: Fabry disease
GD: Gaucher disease
GDPR: General data protection regulation
HCP: Health care professional
LSD: Lysosomal storage diseases
MA: Medical agency
MPS: Mucopolysaccharidoses
PD: Pompe disease
RD: Rare disease
UN: Unmet need
