# Supplementary material for: Understanding the ecosystem of patients with lysosomal storage diseases in Spain: a qualitative research with patients and health care professionals
Source: Orphanet J Rare Dis. 2022 Jan 14;17:17. doi: 10.1186/s13023-021-02168-7 (PMC8760689; doi:10.1186/s13023-021-02168-7)
Supplement: Supplementary file 1 — Additional file 1. Table S1. Pre-Work assignment sent to patients and clinicians prior to the in-depth interview. Table S2. Card Sorting Technique used during the interview with patients. Table S3. Card Sorting Technique used during the interview with clinicians. [file 13023_2021_2168_MOESM1_ESM.docx]

**SUMMARY OF THE FIGURES AND SUPPLEMENTARY TABLES**

- **Table S1:** Pre-Work assignment sent to patients and clinicians prior to the in-depth interview.
- **Table S2:** Card Sorting Technique used during the interview with patients
- **Table S3:** Card Sorting Technique used during the interview with clinicians.
- **Appendix A.** **Patients’ Script:** Summary of patient’s in-depth interview
- **Appendix B. Physicians’ Script:** Summary of clinician’s in-depth interview

**Supplementary Table 1 (Table S1)**

***Pre-Work assignment sent to patients and clinicians prior to the in-depth interview.***

|  |  | **Questions** |
| --- | --- | --- |
| **Patients** | **Vision of the disease** | - How do you feel when you think about FD/GD/PD/MPSI disease? |
|  | **Disease management** | **Pre-diagnosis**   - What were the first symptoms? - When did they appear? - What clinician did you see? - Did you know anything about the disease? - How did you feel at this stage?   **Diagnosis**   - Did they refer you to another specialist? - How long did it take to make the diagnosis? - What was the diagnosis? - How did you feel at this stage?   **Treatment**   - What has been your experience with the treatment? - How do you currently feel about your treatment?   **Follow-up**   - What controls does it carry out? How often? Where do you carry out this follow-up visits? - How do you currently feel at this stage? |
| **Clinicians** | **Priorities in managing patients with LSD** | - What are your therapeutic goals as a physician when treating a patient with FD/GD/PD/MPSI disease? List from highest to lowest importance |
|  | **Patient characteristics and needs** | - How is the attitude of the patient towards the disease? - What are the patient’s expectations about their illness and treatment? - What are the main unmet needs in the diagnosis, treatment, and follow-up phases? |

**Supplementary Table 2 (Table S2)**

***Card Sorting Technique used during the interview with patients***

|  | **Card Sorting 1** | **Card Sorting 2** |
| --- | --- | --- |
| **Patients** | ***Instructions:*** *Below I will show you some cards that show various activities that some patients with your disease do. Please select the actions you do and order them from highest to lowest frequency.* | ***Instructions:*** *Below I will present you some cards that represent some of the aspects that we have pre-identified that can impact on your emotional and physical quality of life. Please select and put a level of importance around your coexistence with the disease from 1 to 10 (1 low importance – 10 high importance)* |
|  | - Social relationships: Change the way you relate to other people - Disconnection: Activities for disconnection like massages, yoga, etc. - Unburden: Talk to someone about the illness - Stop doing things: physical activity, missing work or school, travelling, etc. - Self-Care: Dermatological, aesthetic treatments, etc. - Exercise/physical therapy - Others (diets, etc.) | - Improving the diagnosis. - HCP medical awareness and education. - Coordination between HCP. - Access to treatment. - Ease of administration or monitoring of treatment. - Scientific research. - Information and dissemination about the disease. - Comprehensive approach of patients (physical and emotional). - Specific solutions to improve patient’s quality of life. - Feeling understood by the environment: society, family, administration. |
|  |  |  |
|  |  |  |
|  |  |  |
|  |  |  |

**Supplementary Table 3 (Table S3)**

***Card Sorting Technique used during the interview with clinicians.***

|  | **Card Sorting 1** |
| --- | --- |
| **Clinicians** | ***Instructions:*** *Below I will present you some letters that represent some of the aspects that we have pre-identified that may be relevant around the disease. Please select and put a level of importance around the management of the disease from 1 to 10 (1 low importance – 10 high importance)* |
|  | - Improving the diagnosis. - HCP medical awareness and education. - Coordination between HCP. - Access to treatment. - Ease of administration or monitoring of treatment. - Scientific research. - Information and dissemination about the disease. - Comprehensive approach of patients (physical and emotional). - Specific solutions to improve patient’s quality of life. |
|  |  |
|  |  |
|  |  |
|  |  |
