# Supplementary material for: Understanding the ecosystem of patients with lysosomal storage diseases in Spain: a qualitative research with patients and health care professionals
Source: Orphanet J Rare Dis. 2022 Jan 14;17:17. doi: 10.1186/s13023-021-02168-7 (PMC8760689; doi:10.1186/s13023-021-02168-7)
Supplement: Supplementary file 2 — Additional file 2. Appendix A. Patients’ Script Summary of patient’s in-depth interview: a full description of the questions usedfor the patient’s in-depth interview. [file 13023_2021_2168_MOESM2_ESM.docx]

**APPENDIX A**

***Patients’ Script Summary of patient’s in-depth interview***

**BLOCK 1: INTRODUCTION TO THE INDIVIDUAL**

- How old are you?
- What is your professional status? (active, inactive: unemployed, sick leave, retired)
- Do you have a family? Do you have children or grandchildren? Any other close relatives?
- Who is your biggest support in this disease? Is there anyone in your environment who helps you to cope better with your disease, accompanies you to the doctor, etc.?
- Does any other member of your family also suffer or has suffered from this disease? Who? If so, what relationship do you have?

**BLOCK 2: DIAGNOSIS AND UNDERSTANDING OF THE DISEASE**

**2.1. DIAGNOSIS OF THE PATHOLOGY**

- When did you first develop symptoms of your disease? How many years ago?
- If diagnosed in adulthood:
  - What were the symptoms?
  - What did you do? Did you see a medical specialist? Which one?
  - How long did it take from the time you started having symptoms until you went to this specialist?
  - What happened then? Did they do to you any test? Which one?
  - Did you have to visit any other medical specialist? Which one?
  - Did you have more tests? Which ones?
  - From the time you went to the first doctor, how long did it take to give you a diagnosis?
  - What diagnosis were you given? Was the first diagnosis this disease? Or, on the contrary, were you diagnosed with a different pathology?
  - Which doctor diagnosed you? Was it that first specialist or another one?
  - How did you feel when you were diagnosed with this disease? Why?
- If diagnosed in childhood:
  - Beyond the fact that the diagnosis was made when you were a child, since when have you been aware of having this disease?
  - Who told you about it?
  - Did your life or your relationship with others change at all when you became aware of your pathology? Why? How?
  - When did you first experience symptoms of your disease? At what age? Which were they?
  - How much time passed from the first symptoms to the diagnosis of the disease?
  - What diagnosis were you given? Was the first diagnosis this disease? Or, on the contrary, were you diagnosed with a different pathology?
  - How many specialists do you remember having been visited by?
  - How do you remember your experience with the disease when you were a child? Why?
  - And as a teenager, how do you remember your experience with the disease? Why?

**2.2. UNDERSTANDING AND KNOWLEDGE OF THE PATHOLOGY**

- What did you know about the disease when you were diagnosed?
- At the time of diagnosis, what information did the clinician give you?
- How did you assess this information?
- What type of information did you need or seek at that time, in addition to that provided by the clinican?
- Through which channels? Through which people did you get informed? Did you go to the patients' society?
- How do you value the information you have received over the years? Why?
- How have you been or are you informed on a daily basis about the pathology? Through which media? Through which information channels? (patients' association, specific web pages, clinicians, other specialists, etc.)
- Are you satisfied with the level of information you currently have? Why?
- Do you miss more information about your disease? When? At what times?
- What type of information and what content is most relevant to you as a patient of this disease?

**BLOCK 3: IMPACT OF THE LSD ON DAILY ROUTINES**

**3.1. ATTITUDE AND COEXISTENCE WITH THE PATHOLOGY**

- What does the disease currently represent in your life? What changes has the disease produced or do you think it has produced in you and your environment?
- Right now, how are you feeling emotionally?
- Do you have any fears (living with long-term treatment, incapacity for work, death, genetic transmission, etc.)?
- What are you doing to improve this situation?

**3.2. IMPACT ON THE PATIENT’S LIFE**

- Tell me, what is a normal day like for you?
- On a general level, what are the symptoms of the disease that limit you most in your routines?
- How does the disease affect your day-to-day life as a family? Why?
- And on a professional level, has the disease affected you? If so, how? Why?
- And on a social level, how has the disease affected you? How have your routines been affected? Why?
- **Card Sorting 1**: Below I will show you some cards that show various activities that some patients with your disease do. Please select the actions you do and order them from highest to lowest frequency.
  - Social relationships: Change the way you relate to other people
  - Disconnection: Activities for disconnection like massages, yoga, etc.
  - Unburden: Talk to someone about the illness
  - Stop doing things: physical activity, missing work or school, travelling, etc.
  - Self-Care: Dermatological, aesthetic treatments, etc.
  - Exercise/physical therapy
  - Others (diets, etc.)

**BLOCK 4: ATTITUDE TOWARDS TREATMENT**

**Untreated patient:**

- Do you know the reasons why you are not being treated?
- Were you involved in the decision not to treat?
- Do you know the existing treatments for your disease? Do you know how they work? What is its function?
- How did you find out about it? Who informed you? Through which channels?
- Do you have any doubts about these treatments? Which ones?

**Treated patient:**

**4.1. THE CURRENT TREATMENT**

- What treatment are you currently receiving to treat your disease?
- How long have you been taking/receiving this treatment?
- Do you have any doubts about your treatment? Which ones?
- To what extent do you know the purpose or how the drugs you take work?
- What do you think are the most negative aspects or difficulties in the treatment that could be improved? Why?
- What is the procedure for administering this treatment?
- How often should this treatment be administered?
- In case you attend the hospital,
  - How long does the process of administering this treatment take? How would you rate this time?
  - What about travel time and total hospital stay?
  - Do you feel well cared for during the process?
- If administered at home,
  - Do you use any medication reminders at home? Which ones? Why?
  - Do you prepare in advance for the nursing home visit? Why and how do you do it?
  - How long does the process of administering this treatment at home take?
  - Do you need assistance, beyond that provided by nursing, to administer this medication?
  - What are the main difficulties you encounter? Why?
- At a general level, what do you think are the most negative aspects or difficulties in the treatment that could be improved? (posology, lack of control, etc.) Why?
- Despite these difficulties, what motivates you as a patient to stay with this treatment?

**BLOCK 5: FOLLOW-UP**

**5.1. MONITORING OF THE DISEASE**

- Throughout these years of treatment, how many doctors have you had to see to treat your disease?
- Which professionals are currently helping you in the treatment and control of the disease?
- What is the role of each of them?
- How often do you visit each of them?
- Are you satisfied with your relationship with them? Why?
- Have you had any complications or difficulties in dealing with any of these professionals? With which one? Why?
- Do you think the frequency of visits is adequate? Why?
- Do you feel well attended during the consultation? Why? Do you feel listened to and understood by the professional?

**5.2. INTEGRAL ATTENTION / COMPREHENSIVE CARE**

- What other social-health professionals help you to treat your disease? (nurse, psychologist, dietician, pharmacist, social services, etc.)
- Is their help effective and why?
- What other people help you in the care of your illness? (family, neighbours, friends, colleagues...) How specifically?
- Has your relationship with your environment changed in any way because of your illness? How has it changed?
- At a global level, do you feel understood by your environment? Why?
- If you could ask anything of your (partner, family, friends, colleagues, etc.) in relation to your treatment of the disease or living with the disease, what would it be? Why?

**BLOCK 6: UNMET NEEDS: WAYS OF IMPROVING QUALITY OF LIFE**

- What does having quality of life mean to you?
- How do you rate your quality of life right now?
- **Card Sorting 2**: Below I will present you some cards that represent some of the aspects that we have pre-identified that can impact on your emotional and physical quality of life. Please select and put a level of importance around your coexistence with the disease from 1 to 10 (1 low importance – 10 high importance)
  - Improving the diagnosis.
  - HCP medical awareness and education.
  - Coordination between HCP.
  - Access to treatment.
  - Ease of administration or monitoring of treatment.
  - Scientific research.
  - Information and dissemination about the disease.
  - Comprehensive approach of patients (physical and emotional).
  - Specific solutions to improve patient’s quality of life.
  - Feeling understood by the environment: society, family, administration.
- Why is this value or characteristic important? (address each of the selected values in order of importance)
- Of the selected values, which ones do you think you currently have covered? How? Why?
- And on the other hand, which ones do you have not covered? How could you cover this need? Who could help you with this?
