# Supplementary material for: Understanding the ecosystem of patients with lysosomal storage diseases in Spain: a qualitative research with patients and health care professionals
Source: Orphanet J Rare Dis. 2022 Jan 14;17:17. doi: 10.1186/s13023-021-02168-7 (PMC8760689; doi:10.1186/s13023-021-02168-7)
Supplement: Supplementary file 3 — Additional file 3. Appendix B. Physicians’ Script: Summary of clinician’s in-depth interview: a full description of the questionsused for the physicians’ in-depth interview. [file 13023_2021_2168_MOESM3_ESM.docx]

**APPENDIX B**

***Physicians’ Script: Summary of clinician’s in-depth interview***

**BLOCK 1: THE DISEASE PROFESSIONAL**

**1.1. PROFESSIONAL PROFILE:**

- What is your current professional position and at what center do you practice?
- On a scale of 1 to 10 (1 being very little and 10 being very much), how expert or referent do you consider yourself in the treatment of this disease? Why?
- How long have you been treating the disease?
- Currently, approximately how many patients do you treat per year with this pathology?

**1.2. PATHOLOGY VISION AND PRIORITIES**

- As a doctor, how would you define this disease?
- How would you define the patient profile? What characterizes a patient with this disease (age, sex, lifestyle, other pathologies...)?
- What are the short-term physical, psychological or behavioral consequences of this disease?
- What are your priorities when treating these patients (patient wellbeing, effectiveness of treatments, treatment innovation, patient comfort, being a pioneer, safety...)?
- Do these priorities vary according to patient profile? How? Why?
- What do you think are the patient's expectations about their disease and treatment?
- And your, what are your expectations about the evolution of these patients? Why?

**BLOCK 2: DIAGNOSIS OF THE DISEASE**

**2.1. DIAGNOSIS**

- What is the diagnostic process for this disease usually like?
- Which specialist usually sees the patient first?
- What is the process until the patient comes to you?
- How long does it usually take from the time the patient manifests the first symptoms until the patient receives a diagnosis? Has this time changed over the years?
- Does this time vary depending on the patient profile? (male, female, child/adolescent, youth and adulthood, etc.)
- Which specialist usually diagnoses most patients with this disease?
- What diagnostic toolsor tests do you currently have available?
- How would you rate the level of access to these diagnostic tests?
- What would you say are the main difficulties in diagnosing the disease (lack of knowledge of the pathology by specialists, lack of tests, etc.)?

**2.2. IMPACT ON THE PATIENT**

- Once the diagnosis is made, how do you think patients/caregivers feel when they know they have this disease?
- What information does the patient have about the pathology at the time of diagnosis?
- What would you say are your main fears and why?
- What type of information does the patient demand at this time? What contents?
- Who meets this need for patient information?
- What tools or resources do you provide them with as a doctor to help them live with the disease?

**BLOCK 3. TREATMENT OF THE DISEASE**

- At what point do you as a doctor decide to initiate treatment of a patient with Fabry disease?
- Approximately how long can it take from the time a patient is diagnosed with Fabry disease until they start treatment? Does this time vary based on the patient profile?
- On a general level, what are the main difficulties you face as a doctor during the treatment of Fabry disease patients?
- From the patient's point of view, what do you think are the most negative aspects or difficulties in the treatment that could be improved? (adherence, dosage, etc.) Why?
- What major objectives do you think this pathology should set for the future? Why? (improvement of administration, etc.)

**BLOCK 4. FOLLOW UP**

**4.1 MONITORING OF THE DISEASE**

- What parameters do you consider relevant to monitor in patients with this disease? What tests do you perform for follow-up?
- How often do you visit the patient for follow-up care? What about the other specialists?
- How many healthcare professionals are usually involved in the treatment, follow-up and monitoring of a patient with this disease? Which ones?
- What role does each of them play?
- Do you consider that there is good interaction and communication between specialists?
- What aspects could be improved from your point of view?
- And from the patient's point of view, what aspects could be improved?
- What perception do you think patients have of the medical care received?
- What are the main difficulties for medical specialists when treating patients with the disease? Why?
- Do you consider that the disease is known by other doctors in your specialty? Why? And by specialists in general?

**4.2. INTEGRAL ATTENTION / COMPREHENSIVE CARE**

- What other professionals can help patients and caregivers in the treatment and management of the disease? (psychologist, ...)
- What role does each of them play? How would you say these other professionals complement medical treatment?

**BLOCK 5. UNMET NEEDS: WAYS OF IMPROVING QUALITY OF LIFE**

- What does it mean to you that a patient with this disease has quality of life?
- Despite having this disease, in what aspects could the patient's quality of life be improved?
- What would an improvement in the prescriber-care process mean to you?
- What actions would you consider interesting to improve the management of this disease?
- Below I will present you some letters that represent some of the aspects that we have pre-identified that may be relevant around the disease. Please select and put a level of importance around the management of the disease from 1 to 10 (1 low importance – 10 high importance):
  - Improving the diagnosis.
  - HCP medical awareness and education.
  - Coordination between HCP.
  - Access to treatment.
  - Ease of administration or monitoring of treatment.
  - Scientific research.
  - Information and dissemination about the disease.
  - Comprehensive approach of patients (physical and emotional).
  - Specific solutions to improve patient’s quality of life.
- If you think there is another aspect of great relevance to you, please add it.
- Why are these values or characteristics important? (address each of the selected values in order of importance)
- Of the selected values, tell me, which ones do you usually have covered? Why?
- And on the other hand, which ones are not covered? Who do you think should cover them? How?
